# Does Endometrial Thickness Predict Chemical Pregnancy in Frozen Embryo Transfer? Analysis of 522 artificial cycles performed by a single physician

**DOI:** 10.5935/1518-0557.20260038

**Published:** 2026

**Authors:** Lorena Cristina Santos, Mario Silva Approbato, Alexandre Vieira Santos Moraes, Jane Porfírio Rocha, Gustavo Cardoso Borges, Amanda A. Araujo, Julia Coelho Masiero, Eduardo Camelo de Castro

**Affiliations:** 1 Hospital das Clínicas de Goiás UFG-HC/EBSERH, Goiânia, Goiás; 2 Humana Medicina Reprodutiva, Goiânia, Goiás

**Keywords:** Endometrial thickness (EMT), pregnancy rates, frozen embryo transfer (FET)

## Abstract

**Objective:**

To evaluate whether endometrial thickness (EMT) is associated with pregnancy rates in frozen embryo transfer (FET) cycles.

**Methods:**

This retrospective study analyzed medical records of patients who underwent FET between January 2021 and December 2024. All procedures were conducted by the same physician, using standardized endometrial preparation, with blastocyst-stage transfers. EMT was measured by transvaginal ultrasound on the day of transfer, and pregnancy was confirmed by β-hCG > 25 mIU/ml approximately two weeks later. Statistical analysis included Mann-Whitney U test, linear regression, ROC curve analysis, and chi-square tests, with significance defined as *p*≤0.05.

**Results:**

A total of 522 FET cycles were performed, after exclusions, such as untreated uterine infertility factors and cryopreserved cleavage stage embryos, 478 patients were included with a chemical pregnancy rate of 70.29%. Participants’ median age was 34.9 years (range 23-49). EMT ranged from 3.6 to 20mm (mean 8.82mm). ROC analysis identified 8.3 mm as the optimal cutoff (AUC=0.55; sensitivity 0.542; specificity 0.518). No significant differences were found in pregnancy rates between different EMT intervals (≤8.3mm *vs*. >8.3 mm, *p*=0.4944; <7 mm *vs*. ≥7mm, *p*=0.1036). Regression analysis showed no significant association between EMT and pregnancy (*b*=-1.205; *p*=0.3182; 95% *CI:* -3.914 to 1.504).

**Conclusion:**

In our study, EMT did not significantly predict pregnancy outcomes in FET cycles, indicating that the cancellation of embryo transfers should not be canceled solely on the basis of endometrial thickness.

## INTRODUCTION

Endometrial receptivity and embryo quality are the two main determinants of pregnancy success in IVF/ICSI cycles (Ma *et al.*, 2017) . Despite technological advances, implantation failure still occurs in approximately 30-50% of transfers using high-quality embryos. Implantation failure is considered a distinct and multifactorial process; however, successful embryo implantation is known to require synchronized communication between the embryonic tissue and the maternal endometrium. As such, endometrial receptivity is regarded as a rate-limiting step in in vitro fertilization (IVF) and has garnered increasing attention (Arian *et al*., 2023; Wallach *et al*., 1992).

Among endometrial factors, endometrial thickness (EMT) remains one of the most widely used indicators of receptivity, although its predictive value remains controversial (Liu *et al*., 2025; Lam *et al*., 2022; Liu *et al*., 2018). Some studies associate EMT with improved implantation (Liu *et al.*, 2018; Ma *et al.*, 2017; Noyes *et al.*, 1995) while others report no correlation (Kasius *et al.*, 2014; Lam *et al.*, 2022). Therefore, the present study aimed to evaluate whether EMT measured before progesterone initiation is associated with chemical pregnancy rates in artificial FET cycles conducted by a single physician.

## MATERIAL AND METHODS

This retrospective analysis was performed using all artificial FET cycles performed between January 2021 and December 2024 at a private fertility clinic in Goiânia, Brazil. Dates were extracted from electronic medical records. The study was conducted according to institutional policies on data confidentiality and received a waiver of ethical approval due to its retrospective design.

### Inclusion criteria

(1) patients with indication for Assisted Reproduction treatment who underwent TEC with current endometrial preparation protocols.
(2) patients with cryopreserved blastocyst stage embryos.

### Exclusion criteria

(1) patients with diagnosed and untreated uterine infertility factors such as septate uterus, intrauterine adhesions, adenomyosis, and endometrial polyps. Endometrial thickness was not used as a criterion for cycle cancellation.
(2) patients with cryopreserved cleavage stage embryos.

Since this study did not involve therapeutic interventions or modifications to standard IVF-TEC protocols, no additional approval from the institutional ethics committee was required.

### Endometrial preparation

Endometrial preparation consisted of oral administration of estradiol valerate starting on the second or third day of menstruation. This regimen stimulated endometrial proliferation and thickening and aimed to achieve a thickness of 7 mm, measured by transvaginal ultrasound. In cases of suboptimal endometrial response, in addition to increasing the dose, transdermal estradiol was added. Approximately 10 to 16 days after estradiol administration, vaginal micronized progesterone was started to prepare the endometrium for embryo transfer.

Serum progesterone levels were measured approximately 24 hours before embryo transfer, approximately 6±2 hours after the last vaginal dose of 200 mg of micronized progesterone. Results were available on the same day.

Patients with serum progesterone levels <10 ng/mL received an additional subcutaneous dose of 25 mg progesterone (Progestan Dex; Kocak, Turkey) once daily for at least 42 days, starting on the day of embryo transfer. This supplemental dose was administered at the same time each day, coinciding with the time of the first injection. Luteal phase support was continued until pregnancy was confirmed or discontinued on the day of the pregnancy test if it was negative.

### Endometrial Assessment

The endometrial assessment protocol remained consistent throughout the study period. Endometrial thickness (EMT) was measured by the second author, a medical specialist at the fertility center, using two-dimensional transvaginal ultrasonography. EMT was defined as the maximum distance between the echogenic interfaces of the myometrium and endometrium and was measured in the midsagittal plane on the day before starting micronized progesterone.

Endometrial morphology was classified into two main patterns: (1) a trilaminar (three-line) pattern, characterized by two outer hyperechoic lines representing the endometrial-myometrial interface on the anterior and posterior uterine walls, and two inner hypoechoic layers separated by a central hyperechoic line representing the interface between the endometrial surfaces; and (2) a homogeneous echogenic pattern. EMT referred to the peak thickness measured during the estradiol phase (E2).

### Thawing and Hormonal Support

Embryos were thawed using the Vit Kit - Warm (FUJIFILM Irvine Scientific, USA), and between one and three embryos were transferred into the uterus using a Guardian Access ET catheter (Cook Incorporated, IN, USA). The best frozen blastocysts from each patient were selected for thawing. After embryo transfer, hormonal supplementation with progesterone was continued for 14 days until a blood pregnancy test was performed. Patients with a positive result continued hormonal supplementation until the 12th week of gestation.

### Cycle Results

Chemical pregnancy was confirmed by β-hcg testing (>25 mIU/ml) approximately two weeks after transfer.

### Statistical Analysis

Data were analyzed using BioEstat software (Belém-PA, Brazil). All tests were two-tailed and *p*<0.05 was considered statistically significant. Descriptive data are presented as median (range), mean ± standard deviation (SD). Patients were divided into two groups: those who achieved a chemical pregnancy (Group A) and those who did not (Group B). A statistical analysis using the Mann-Whitney U test was performed to assess the association between endometrial thickness and pregnancy rates. Regression analysis was performed to assess the association between endometrial thickness and pregnancy rates. To evaluate the ideal cutoff point for endometrial thickness in predicting gestational outcomes, the ROC (Receiver Operating Characteristic) curve was used and the endometrial thickness cut-off was performed and statistical comparisons were made by the chi-square test (χ^2^). Additionally, based on scientific evidence suggesting that 7 mm as the minimum threshold for optimal endometrial receptivity, patients were categorized into two groups: those with a thickness <7 mm and those with a thickness ≥7 mm and statistical comparisons performed by the chi-square test (χ^2^).

## RESULTS

**Figure 1:**
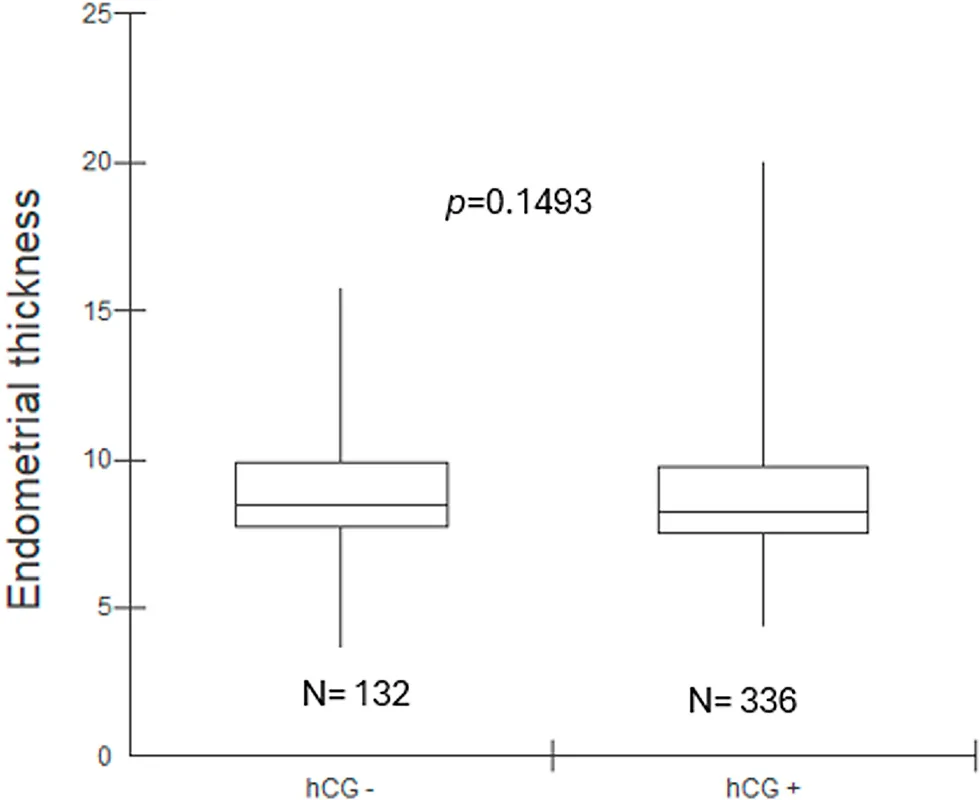
Distribution of patients according to endometrial thickness and hCG result. Lab-Rep-UFG/Humana Medicina Reprodutiva (2021-2024).

Between January 2021 and December 2024, a total of 522 FET cycles were performed with cumulative pregnancy rate 71,68%; 44 patients were excluded: seventeen because of exclusion criteria and twenty-seven for age matching, resulting in a final sample of 478 patients. The median age was 34.9 years (range, 23-49) and the cumulative chemical pregnancy rate (PR), after the exclusion criteria, was 70.29%. EMT ranged from 3.6 to 20 mm (mean 8.82±2.4 mm). No statistically significant difference was observed between the groups (Figure 1).

Mann-Whitney (U=22421.50)

**Figure 2:**
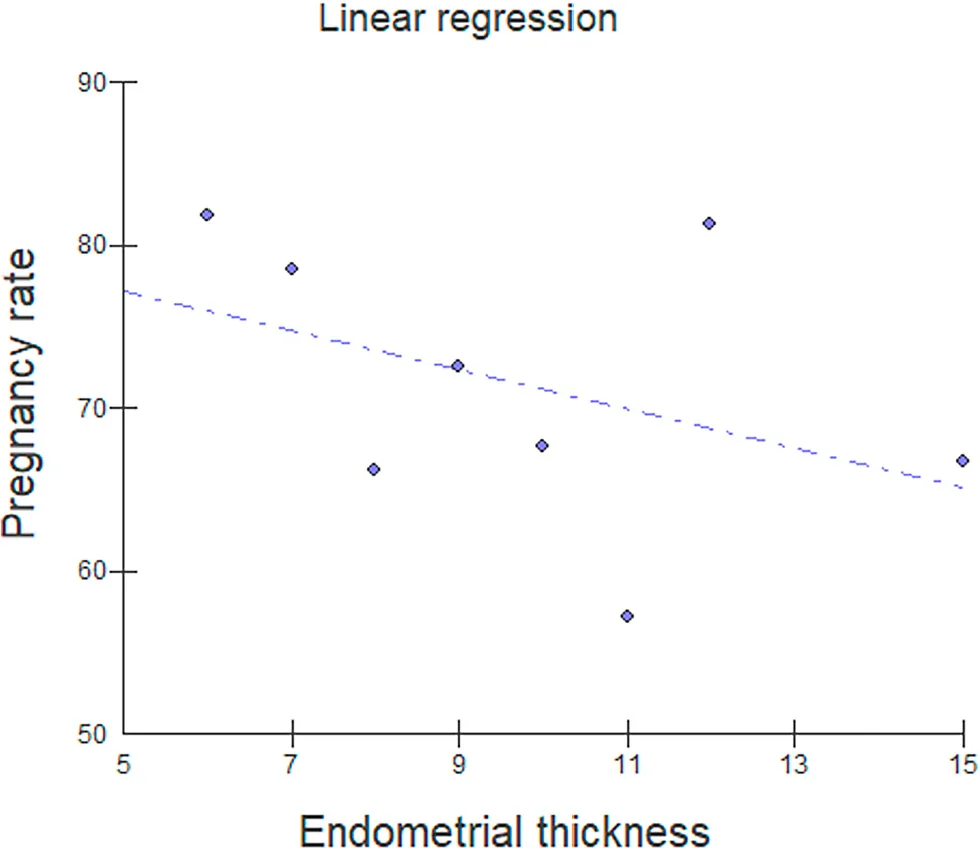
Endometrial thickness linear regression curve. Lab-Rep-UFG/Humana Medicina Reprodutiva (2021-2024).

A simple linear regression analysis was performed to evaluate the relationship between the endometrial thickness and pregnancy rates. The model was not statistically significant (*F*(1,6)=1.18; *p*=0.319), indicating no significant linear association between the variables. The coefficient of determination (*R^2^*=0.1649) showed that only 16.5% of the variance in the PR was explained by the EMT. The regression coefficient was not significant (*b*=-1.205; *p*=0.3182; 95% *CI:* -3.914 to 1.504), suggesting that EMT variations do not significantly predict variations in the PR (Figure 2).

**Figure 3:**
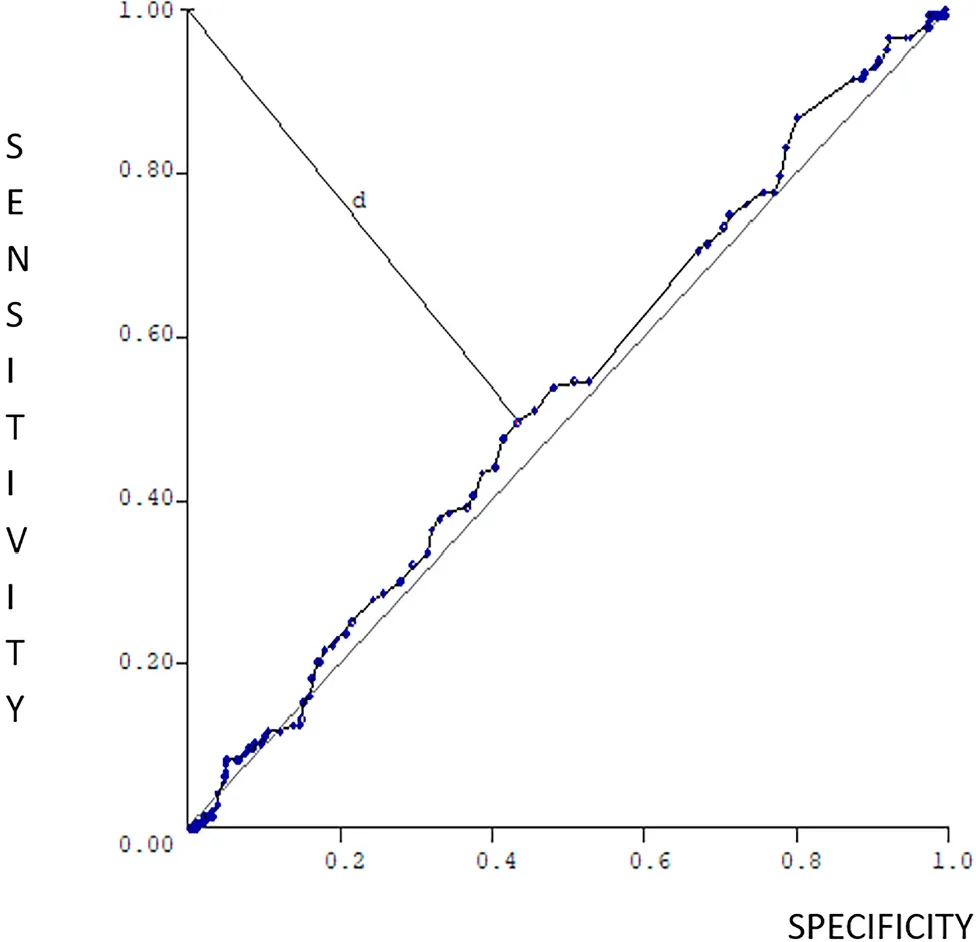
Endometrial thickness ROC curve. Lab-Rep-UFG/Humana Medicina Reprodutiva (2021-2024).

To assess the endometrial thickness cut-off, a ROC curve analysis was performed (Figure 3) with an area under the curve (AUC)=0.55 and sensitivity and specificity values of 0.542 and 0.518, respectively. Based on the ROC analysis, an optimal cut-off value of 8.3 mm was identified.

Cut-off: 8.3

Distance (d): 0.665

Based on cut-off, patients were subsequently stratified into two groups: Group 1 with endometrial thickness≤8.3 mm and Group 2 with thickness > 8.3 mm. No statistically significant difference in pregnancy rates was observed between the groups (*p*=0.4944) as shown in Table 1.

**Table 1:** Pregnancy rates below and above 8.3 mm endometrial thickness

Endometrial thickness on day of HCG	BHCG positive (%)	BHCG negative (%)	p-value^a^
≤8.3 >8.3	72.40 (181/250) 67.98 (155/228)	27.60 (69/250) 32.02 (73/228)	0.4944
Total	336	142	

a Chi-square (*p*<0.05)

Considering scientific evidence that supports 7 mm as the minimum threshold for optimal endometrial receptivity, a chi-square test was performed to compare pregnancy rates between two groups stratified by endometrial thickness: < 7 mm and ≥ 7 mm (Table 2).

**Table 2:** Pregnancy rates below and above 7.0 mm endometrial thickness

Endometrial thickness on day of HCG	BHCG positive (%)	BHCG negative (%)	p-value^a^
<7.0 ≥7.0	79.24 (42/53) 69.17 (294/425)	20.76 (11/53) 30.83 (131/425)	0.1036
Total	336	142	

a Chi-square (*p*<0.05)

In this study population, no statistically significant difference in pregnancy rates was observed between the groups (*p*=0.1036).

## DISCUSSION

The relationship between EMT and pregnancy outcomes has long been a subject of debate. Despite numerous meta-analyses and large-scale studies, the controversy remains unresolved. Notably, there is a significant discrepancy between the findings of prospective and retrospective studies.

Many authors have reported significant differences in pregnancy rates above and below threshold EMT values ranging from 7 to 10 mm (Liu *et al*., 2025). Several studies suggest that an adequately thick endometrium is associated with improved implantation potential, whereas thin endometrial linings are often correlated with reduced implantation rates and lower pregnancy success (Tomic *et al.*, 2020). Conversely, excessively thick endometria have also been linked to unfavorable reproductive outcomes (Xu *et al*., 2022). Our findings suggested that different EMT intervals revealed no statistically significant differences, suggesting that EMT was not a reliable predictor of pregnancy in this study population (Lam *et al.*, 2022; Noyes *et al.*, 1995; Shakerian *et al.*, 2021; AYUSTAWATI *et al.*, 2002).

Some studies report a linear positive association between EMT and pregnancy outcomes (Al-Lamee *et al.*, 2024; Liao *et al.*, 2022), while others propose a nonlinear relationship (Lam *et al.*, 2022; Shakerian *et al.*, 2021). Inconsistent findings, reliance on arbitrary cutoff values (e.g., <6, <7, or <8 mm) and a lack of standardized definitions further complicate the understanding of EMT’s role in predicting pregnancy outcomes. Despite various cutoff values have been proposed to define a “thin” endometrium, with most studies setting this threshold at less than 7-8 mm on the day of ovulation trigger. However, reported cutoff values have ranged from < 6 mm (Mathyk *et al.*, 2023) to >14 mm (El-Toukhy *et al.*, 2008).

Our findings suggest that EMT alone may not be a reliable predictor of pregnancy in artificial FET cycles. This aligns with recent meta-analyses reporting weak or no correlation between EMT and live birth rates (Lam *et al.*, 2022; Shakerian *et al.*, 2021). Although a minimal threshold of 6-7 mm is often cited, our data indicate satisfactory pregnancy outcomes even in patients with thinner linings.

In contrast, a study involving 743 frozen-thawed embryo transfers using autologous embryos found a lower pregnancy rate in women with an EMT between 7 and 8 mm compared to those with an EMT greater than 8 mm (Martel *et al.*, 2021). Similarly, El-Toukhy *et al.* (2008) analyzed 768 consecutive medicated frozen embryo replacement cycles and demonstrated that EMT between 9 and 14 mm was associated with higher implantation and pregnancy rates compared to ET of 7-8 mm. Ning-Zhao Ma and collaborators (Ma *et al.*, 2017) stratified patients into three groups based on EMT (group A: ≤8 mm; group B: 9-14 mm; group C: ≥15 mm) and observed that live birth rate, clinical pregnancy rate, early miscarriage rate, and ectopic pregnancy rate were all influenced by EMT at the day of hCG administration, with outcomes improving as EMT increased.

National data from autologous in IVF and FET cycles in Canada showed that live birth rates plateau after an EMT of 7-10 mm. However, EMT below 6 mm was clearly associated with significantly reduced live birth rates in both fresh and frozen embryo transfer cycles (Mahutte *et al.*, 2022). Interestingly, in our study, patients with EMT ≤6 mm still exhibited good pregnancy rates, this suggests that, in the present analysis, endometrial thickness did not play a determining role in pregnancy outcomes.

As previously discussed, several studies have suggested that EMT below certain cut-off negatively predicts pregnancy, often leading to cancellation or postponement of embryo transfer cycles. However, cut-off values commonly used to define a thin endometrium are largely arbitrary and lack robust biological justification. As a result, the determination of an optimal threshold remains uncertain, thereby complicating comparisons across studies. Current evidence regarding the relationship between EMT and pregnancy outcomes remains conflicting and insufficient to justify definitive clinical decisions. Such decisions carry significant clinical, financial, and ethical implications (Shakerian *et al.*, 2021). Our data indicate EMT alone may not be a reliable predictor of pregnancy rates and that “thin” endometrium can still be associated with good pregnancy rates. Therefore, the common clinical practice of canceling embryo transfers when EMT is less than 7 mm may be unwarranted, as it potentially denies patients the opportunity for pregnancy. Nevertheless, confounding variables such as embryo quality, serum progesterone, and endometrial morphology were not controlled, which may influence outcomes. Prospective studies are needed to clarify whether EMT independently affects implantations.

## CONCLUSION

In conclusion, endometrial thickness was not identified as a significant predictor of chemical pregnancy in FET cycles. Our data indicate that embryo transfers should not be canceled solely based on EMT measurements, as favorable outcomes can occur even in cases with thinner endometria.
